# Gene Flow of a Forest-Dependent Bird across a Fragmented Landscape

**DOI:** 10.1371/journal.pone.0140938

**Published:** 2015-11-18

**Authors:** Rachael V. Adams, Theresa M. Burg

**Affiliations:** University of Lethbridge, Department of Biological Sciences, Lethbridge, Alberta, Canada; Australian National University, AUSTRALIA

## Abstract

Habitat loss and fragmentation can affect the persistence of populations by reducing connectivity and restricting the ability of individuals to disperse across landscapes. Dispersal corridors promote population connectivity and therefore play important roles in maintaining gene flow in natural populations inhabiting fragmented landscapes. In the prairies, forests are restricted to riparian areas along river systems which act as important dispersal corridors for forest dependent species across large expanses of unsuitable grassland habitat. However, natural and anthropogenic barriers within riparian systems have fragmented these forested habitats. In this study, we used microsatellite markers to assess the fine-scale genetic structure of a forest-dependent species, the black-capped chickadee (*Poecile atricapillus*), along 10 different river systems in Southern Alberta. Using a landscape genetic approach, landscape features (e.g., land cover) were found to have a significant effect on patterns of genetic differentiation. Populations are genetically structured as a result of natural breaks in continuous habitat at small spatial scales, but the artificial barriers we tested do not appear to restrict gene flow. Dispersal between rivers is impeded by grasslands, evident from isolation of nearby populations (~ 50 km apart), but also within river systems by large treeless canyons (>100 km). Significant population genetic differentiation within some rivers corresponded with zones of different cottonwood (riparian poplar) tree species and their hybrids. This study illustrates the importance of considering the impacts of habitat fragmentation at small spatial scales as well as other ecological processes to gain a better understanding of how organisms respond to their environmental connectivity. Here, even in a common and widespread songbird with high dispersal potential, small breaks in continuous habitats strongly influenced the spatial patterns of genetic variation.

## Introduction

Dispersal and gene flow play an important role in maintaining the genetic and functional connectivity of populations; a process necessary for species’ persistence. However, it has long been recognised that variation within the landscape matrix separating habitat patches can influence the transition stage of individuals across a landscape [1]. Consequently, a landscape which impedes dispersal can break down population (and functional) connectivity and overtime lead to isolation, population genetic differentiation and divergence. Landscape genetics now offers a framework to explicitly test the effects of landscape features and environmental variables on spatial patterns of genetic differentiation; providing a means to identify factors either facilitating or impeding gene flow among populations [2–4].

Some landscapes that are spatially heterogeneous can affect movement of organisms among resource patches as well as the pattern of dispersal, and in turn influence gene flow and population dynamics [5]. In such landscapes, suitable habitat is not always continuous but is patchily distributed, and gaps between patches can vary in size. In addition, patches themselves can differ in their quality. For example, two patches may experience different levels of food resources, predation and reproductive opportunities, which influences an organisms’ decision to disperse or not. However, the impact of fragmentation is ultimately from the species viewpoint and can affect some species without affecting others. Nevertheless, a myriad of studies exist on the effects landscape heterogeneity has on movement and subsequent genetic structure in a number of organisms [6].

One example of a heterogeneous landscape is the Great Plains in North America, a broad area of flat land found east of the Rocky Mountains and west of the Missouri River. The landscape is dominated by prairie, steppe, and grassland; with forested areas restricted to riparian zones. These zones are situated adjacent to streams, rivers, lakes and wetlands are among the most valuable, productive and structurally diverse landscapes [7–9]. This naturally rich environment provides unique habitat for wildlife [10]. In western North America, riparian ecosystems along river flood plains are dominated by poplar trees (*Populus* spp.) [11,12] whereas the surrounding landscape is dominated by treeless prairie grassland. As such, riparian ecosystems are the only wooded areas in the northern Great Plains providing critical habitat and dispersal corridors for forest-dependent organisms [13]. More importantly, riparian zones have been shown to reverse the effects of habitat fragmentation by enhancing connectivity and facilitating individual movement between areas that would otherwise become isolated [14,15]. However, even within these limited forested regions, the quality and structure of the environment can vary both spatially (e.g., upstream vs. downstream) and temporally (e.g., diversion of rivers). As such, both natural and human-mediated processes can further impact the pattern of habitat fragmentation of patchily distributed resources in a heterogeneous landscape.

River management can have long-lasting, negative impacts on riparian species. Urbanisation and increasing demand for water for agriculture, industrial and domestic use have resulted in 82% of large rivers (> 1000 km) across North America being dammed and/ or diverted [16]. Changes to river flows and modifications to associated habitat can also affect the health of riparian ecosystems. For example, a decline in riparian forests has been observed downstream from major dams such as the Truckee River, Nevada [17], the Marias River, Montana [18] and the Oldman River [19] and Willow Creek, Alberta [20]. All studies found healthier forests upstream than downstream. The effects can be reversed through restoration efforts [17], but without these efforts, fragmentation of riparian habitats through human-mediated processes could lead to drastic reductions in population size or local extinctions particularly of riparian specialist species.

Not only is there concern over riparian forest decline, these riparian habitats can also provide unique zones of ecological transitions. Within river systems, the distributions of riparian poplars can overlap resulting in hybrid zones. These hybrid poplar zones can dramatically impact riparian biodiversity and habitat complexity [11,21]. As a result, studies have found that hybrid poplar zones have higher arthropod abundance such as the poplar bud gall mite [22] and gall producing aphids [23] which can affect the distribution of nesting birds, bird abundance [24], arthropod speciation [25] and species richness [13,26,27].

Riparian woodlands are important areas for breeding, wintering and migrating birds providing corridors through areas of unsuitable habitat (e.g., deserts and grasslands). Loss of riparian woodland could have a negative impact on organisms throughout large portions of their range. A number of studies have documented the distribution, density and diversity of riparian bird species [28,29] and their response to riparian woodland fragmentation [15,30–33]. The effects of changes in riparian habitats on the distribution of genetic variation, however, are less well studied in birds, perhaps because their dispersal capabilities suggest that gene flow would be unaffected. Studies on genetic differentiation of terrestrial [34–38] and aquatic plants [39] as well as other aquatic organisms such as fish [40–42], amphibians [43] and invertebrates [44,45] in riparian systems are comparatively more common. These studies show that fragmentation of riparian woodland can have evolutionary effects, such as increased population differentiation as a result of reduced dispersal and isolation. A similar response may be observed in other riparian organisms, and therefore conservation and management of these ecosystems is critical.

With growing concern over global anthropogenic change, it is important to understand the influence of landscape features on dispersal, gene flow and population connectivity across heterogeneous landscapes. The Great Plains offers a unique and valuable study area for testing the effects of spatial heterogeneity on gene flow of the black-capped chickadee, a forest-dependent species. The black-capped chickadee (*Poecile atricapillus*), a common songbird to North America [46], is an ideal model organism for understanding the ecological state of riparian ecosystems because it responds relatively quickly to environmental change [47]. Despite being a resident species, black-capped chickadees are capable of short distance dispersal within areas containing sufficient forest cover [48]. In the Great Plains, dispersal is dependent on forested riparian corridors, but within riparian areas, features such as reservoirs or degraded woodland may impede dispersal and subsequent gene flow both within and between river systems. Understanding how variation in riparian corridors influences functional connectivity in chickadees will bridge the gap in our knowledge of species’ ecology and offer insights into the significance of these ecosystems for movement and species preservation.

The aim of this study was to assess the genetic diversity, population structure and genetic differentiation of the black-capped chickadee across multiple river drainages in a small region of the Great Plains. Genetically distinct populations have previously been identified in this species on a large geographical scale [49–52], so we predict that additional substructuring will be observed on a smaller spatial scale. As well as testing for the effect of geographical distance on gene flow, we also investigated the influence of landscape features, including land cover, elevation and hybrid poplar zones, on the observed population genetic differentiation (an indirect measure of gene flow) using a landscape genetics approach. For land cover, where continuous riparian forest should act as dispersal corridors facilitating dispersal and gene flow within river systems, we predict that large gaps in woodland will act as barriers to dispersal and gene flow and lead to population differentiation within a river system. Between river systems, we predicted that prairie grassland would restrict dispersal and lead to population differentiation. Black-capped chickadees are not found at high elevations therefore we also predicted that the Rocky Mountains would restrict dispersal between rivers systems in the western Great Plains. As changes in elevation are associated with variation in land cover, we combined the two variables to generate two alternative hypotheses. First, a transition from low elevation mixed/ deciduous forest in the east to high elevation coniferous forest in the west, combined with displacement by other chickadees (e.g., mountain chickadee *P*. *gambeli*) at high elevations, would restrict dispersal resulting in increased genetic differentiation in the west. Alternatively, the more densely forested areas in the foothills of the Rocky Mountains may facilitate dispersal. Finally, we predicted reduced dispersal in hybrid poplar zones within riparian corridors. These zones harbour diverse insect communities which are prey for black-capped chickadees, and may attract chickadees in large numbers; reducing dispersal.

## Materials and Methods

### Study Area

**Fig 1:**
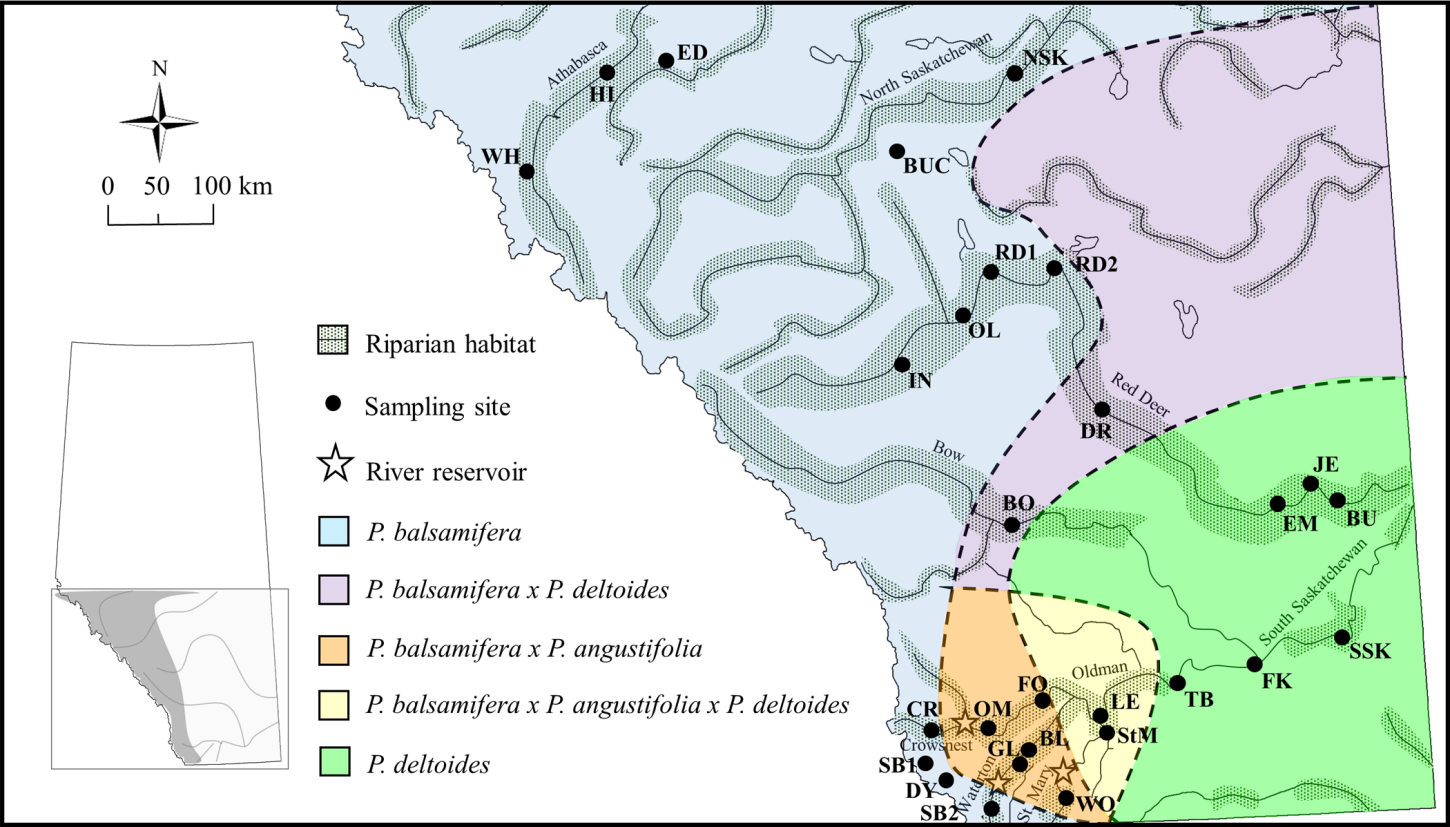
Map showing sampling locations, barriers and hybrid poplar zones within riparian habitats. Map of Southern Alberta illustrating riparian woodlands within each river system (shaded dots), sampling locations of the black-capped chickadee *Poecile atricapillus* (black dots; see Table 1 for abbreviations) and artificial barriers (i.e., river reservoirs represented as stars). Approximate boundaries of pure and hybrid poplar zones (not to scale) are denoted by the dashed lines and corresponding colours (see legend).

Southern Alberta, located within the northern Great Plains, is a highly heterogeneous landscape characterised by densely forested montane habitat in the west (Rocky Mountains), transitioning to a narrow zone of aspen parkland and then quickly to prairies, dominated by temperate grasslands. Within the prairies, forested areas are restricted to riparian ecosystems within river systems which flow throughout the landscape. Both naturally treeless river canyons and artificial reservoirs exist along the river systems, resulting in a patchy woodland corridor (Fig 1). In our study, four species of riparian poplar occur: narrowleaf cottonwood (*Populus angustifolia*), balsam poplar (*P*. *balsamifera*) and the closely related black cottonwood (*P*. *tricocarpa*), and prairie or plains cottonwood (*P*. *deltoides*). These four species hybridize to provide globally-unique hybrid zones [13,22] (Fig 1) that supports diverse insect communities [53]. The study area also encompasses a continuous elevational gradient going from high elevation in the west to low elevation in the east.

### Sample collection

Birds were captured using mist nets and call playback. Each individual was banded with a uniquely numbered band and blood samples (< 100 μl) were taken from the brachial vein (S1 Table). Using a transect-based sampling approach, we aimed to sample 20 individuals from each location (or population) along 10 river systems and one creek in Southern Alberta (Table 1). Each sampling site was confined to a 10 km radius where possible and geographic location was recorded for each site. Sampling sites were chosen strategically to include areas on either side of natural and man-made barriers, different river drainages and riparian habitat with different species of poplars. Samples from our previous study [52] were incorporated to cover additional river systems (i.e., CAB along the North Saskatchewan and Athabasca Rivers, SAB1 on the Castle River, and SAB2 on the Belly River). Sampling took place over eight breeding seasons (2007–2014) with most of the samples collected in 2013 and 2014. All the necessary permits (Government of Alberta Collection License, Canadian Wildlife Service (CWS) Prairies, Provincial Parks and Alberta Parks) and permissions (City and Municipal parks, private landowners) were applied for and approved prior to fieldwork.

**Table 1:** Sampling location information.

Pop.	Abbrev.	Associated river system	Lat (°N)	Long (°W)	*N*	PA	*Ho*	*He*	AR	*F*_IS_
Whistler	WH	Athabasca River	52.8491	118.0797	1	0	-	-	-	-
Edson	ED	Athabasca River	53.6286	116.8019	1	0	-	-	-	-
Hinton	HI	Athabasca River	53.3936	117.5843	2	0	-	-	-	-
Buck Lake	BUC	North Saskatchewan River	52.9721	114.6046	7	2	0.714	0.633	4.18	-0.122
Edmonton	NSK	North Saskatchewan River	53.4974	113.5357	23	3	0.683	0.676	4.68	0.001
Olds	OL	Red Deer River	51.7637	114.4128	4	1	-	-	-	-
Innisfail	IN	Red Deer River	52.0415	113.9703	9	0	0.616	0.643	4.33	0.111
Red Deer 1	RD1	Red Deer River	52.3135	113.7858	18	0	0.564	0.660	4.51	0.204
Red Deer 2	RD2	Red Deer River	52.3376	113.1258	19	3	0.699	0.697	4.60	0.012
Drumheller	DR	Red Deer River	51.4609	112.7258	20	1	0.667	0.708	4.60	0.072
Emerson Bridge	EM	Red Deer River	50.9161	111.9007	4	0	-	-	-	-
Jenner	JE	Red Deer River	50.844	111.1527	2	0	-	-	-	-
Buffalo	BU	Red Deer River	50.8494	110.697	1	0	-	-	-	-
Wyndam-Carseland PP	BO	Bow River	50.829	113.422	20	2	0.648	0.678	4.57	0.051
Southern Alberta 2	SB2	Waterton River	49.0694	113.8561	29	2	0.643	0.676	4.54	0.059
Drywood Creek	DY	Drywood Creek	49.2978	114.0225	20	0	0.618	0.678	4.66	0.103
Southern Alberta 1	SB1	Castle River	49.3908	114.3397	30	5	0.659	0.649	4.29	-0.006
Crowsnest	CR	Crowsnest River	49.574	114.2405	20	2	0.698	0.708	4.79	0.004
Oldman River Reservoir	OM	Oldman River	49.5584	113.821	10	1	0.637	0.675	4.58	0.061
Blue Trail Park	BL	Waterton River	49.4295	113.4961	4	0	-	-	-	-
Glenwood	GL	Waterton River	49.4019	113.5933	3	1	-	-	-	-
Fort Macleod	FO	Oldman River	49.7328	113.399	15	1	0.631	0.641	4.21	0.001
Lethbridge	LE	Oldman River	49.696	112.8633	48	10	0.611	0.654	4.34	0.094
St.Mary	StM	St Mary River	49.5891	112.8889	5	2	-	-	-	-
Woolford PP	WO	St Mary River	49.175	113.1876	3	1	-	-	-	-
Taber	TA	Oldman River	49.8133	112.1701	4	0	-	-	-	-
Forks	FK	Oldman/ Bow/ S.Sask confluence	49.9249	111.6908	1	0	-	-	-	-
Medicine Hat	SSK	South Saskatchewan River	50.0412	110.6631	20	1	0.644	0.667	4.01	0.068

Population name (Pop.), site abbreviation (Abbrev.), location (latitude (Lat) and longitude (Long)), sample size (*N*) and microsatellite summary statistics for each population across all loci: number of private alleles (PA), observed (*Ho*) and expected (*He*) heterozygosities, allelic richness (AR) and inbreeding coefficients (*F*_IS_).

#### Ethics Statement

Sampling was conducted and approved under the University of Lethbridge Animal Welfare Protocol No. 1028 in accordance with the Canadian Council on Animal Care Regulations.

### Genetic diversity and population structure

DNA extraction, amplification and genotyping were performed on all individuals following the procedures described in [52]. Twelve polymorphic microsatellite loci were used for DNA amplifications (PAT MP-14, PAT MP-43, Escu6, Titgata39, Titgata02, CcaTgu11, Cuμ28, PmanTAGAn71, Ase18, VeCr05, CtC101 and Pij02; S2 Table). For Pij02, the two-step annealing temperatures were adjusted to 52°C and 54°C. The one individual genotyped for ≤ 5 loci and three birds from known or suspected family groups (i.e., caught at the same time, showed patterns consistent with family groups at multiple loci) were removed from analyses.

Errors within the data (e.g., input errors, allelic dropout, stutter and null alleles) were assessed in MICRO-CHECKER v2.2 [54]. To assess the level of genetic diversity, allelic richness was calculated in FSTAT v2.9.2.3 [55], and observed and expected heterozygosities and inbreeding coefficients were calculated in GenAlEx v6.5 [56]. Tests for deviations from Hardy-Weinberg equilibrium (HWE) and linkage disequilibrium (LD) were performed in GENEPOP v4.0.10 [57,58] using default Markov chain parameters (100 batches, 1000 iterations and 1000 dememorisation steps). Significance was tested using a modified False Discovery Rate (FDR) correction method [59].

Populations with ≤ 5 individuals were removed from population based analyses. Genetic structure was quantified for all pairwise combinations of populations using *F*_ST_ implemented in GenAlEx v6.5 to assess the level of population genetic differentiation. To complement the conventional *F*-statistic, we calculated an additional pairwise estimate of genetic differentiation (*D*_EST_) in SMOGD v1.2.5 [60,61] and standardised *F*’_ST_ in GenAlEx v6.5 and significance was tested by the FDR correction method. To further assess population genetic structure we carried out a hierarchical analysis of molecular variance (AMOVA) in ARLEQUIN v3.5 [62]. A basic model of isolation by distance (IBD), following Rousset’s transformation [63], was then conducted using a Mantel test in IBDWS v. 3.2.3 [64] with 10,000 permutations to evaluate the effects of Euclidean geographic distances on population connectivity.

### Genetic clustering analyses

To validate pairwise estimates of genetic differentiation, we explored the number of genetic groups within the study system using an individual based Bayesian clustering method, STRUCTURE v2.3.4 [65], as well as a non-Bayesian exploratory clustering method, Discriminant Analysis of Principal Components (DAPC) [66]. STRUCTURE identifies the most likely number of genetic clusters (*K*) by assigning individuals to groups while maximising HWE and minimising LD. All individuals were included as assignments are based on individual multilocus genotypes and STRUCTURE was run with the admixture model, correlated allele frequencies [67] and locations as priors (locpriors). Ten independent runs (50,000 burn in followed by 200,000 McMC repetitions) were conducted for each value of *K* (1–10) to infer the optimal number of clusters (*K*). Results were averaged and the true *K* was determined using STRUCTURE HARVESTER v0.6.6 [68] from both delta *K* (Δ*K*) [69], and mean log likelihood LnPr(X|K). Any individuals showing mixed ancestry (e.g., 50% to cluster 1, and 50% to cluster 2) were rerun to determine correct assignment. If individuals from multiple populations assigned to the same genetic cluster, a hierarchical analysis was carried out to test for additional substructure within those clusters using the same parameters as the initial run, but only five runs for each *K* value.

In addition to assessing population genetic structure across the whole study area, we were also interested in determining whether populations located on either side of a potential barrier were genetically distinct. If so, this would provide an indication of restricted gene flow. Populations of interest include those separated by an extensive break in riparian woodland (e.g., LE, SSK and BO (Fig 1)) and those separated by artificial structures (e.g., CR and OM separated by the Oldman Reservoir, and SB2 and GL/BL by the Waterton Reservoir (Fig 1)). Populations StM and WO are separated by both an artificial (St. Mary Reservoir) and natural (gap in woodland) structures. Prior to the establishment of the St. Mary Reservoir, this river system was composed of sparsely distributed poplar woodland [70], however, the reservoir has since had a negative impact on downstream riparian woodland, leading to the complete loss of woodland [71]. STRUCTURE was run using the same parameters as the previous run (five replicates for each *K* with 50,000 burn in, 200,000 McMC) for each pair of populations separated by a “barrier”. This method also removes noise present from additional data and allows the determination of population structuring at very small spatial scales.

DAPC is a multivariate method implemented in the program R v 3.1.3 [72] using the package ADEGENET [73] designed to identify and visualise diversity among groups without using geographical information [66]. As such, it allows us to test population differentiation without an *a priori* assumption on groupings. Unlike STRUCTURE, DAPC does not assume Hardy-Weinberg or linkage equilibrium. For DAPC analysis (function dapc), first a principal component analysis (PCA) is performed on predefined populations (i.e., sampling site) where the genotypic data are transformed into principal components. The PCA variables are then used in the discriminant analysis (DA). This initial PCA step ensures that no correlated variables are input into the DA and that a weighted and reduced number of variables are included; 50 principal components (PCs) were retained corresponding to > 85% of the variance. DAPC defines groups by minimising within group variation and maximising among group variation.

### Parameterization of landscape variables

Landscape variables were selected based on two model hypotheses, isolation by distance (IBD) which assumes spatial homogeneity [74] and isolation by resistance (IBR) [75] which assumes spatial heterogeneity. Small populations (≤ 5 individuals) were excluded from analyses. Pairwise resistance distances were calculated for different landscape variables (or maps) using a circuit model of landscape connectivity in CIRCUITSCAPE v4.0 [75]. This model calculates all possible pathways of least resistance to gene flow using circuit theory and allows for multiple landscape features to be tested. First, a uniform resistance landscape map was created and pairwise distances were calculated to represent the null model of IBD (i.e., all pixels assigned a cost value of 1). An additional four landscape variables were then chosen for parameterization, and were reclassified with a 100 m resolution to represent hypothetical resistance values to dispersal in ArcMap ESRI (Table 2). Categorised land cover and topographical maps from GEOBASE (www.geobase.ca) were then reclassified to represent three separate hypothesised resistance maps; land cover, elevation and land-elevation, with the latter accounting for the influence of both land cover and elevation combined. Finally, to determine if hybrid poplar zones influence chickadee dispersal and gene flow, we created an additional hypothesised resistance map, ‘hybrid’ (Table 2). For hybrid zone based models and analyses, only the 12 populations sampled within rivers associated with hybrid zones (Drywood Creek and the Red Deer, Oldman, Crowsnest, Waterton, St. Mary and South Saskatchewan Rivers) were included. All resistance maps were clipped in ArcMap while retaining a buffer around the study area to leave enough landscape available for dispersal and to prevent edge effects [76].

**Table 2:** Information for each landscape variable tested including resistance level(s), hypothesis and corresponding predictions.

Landscape Variable	High/low resistance	Hypothesis	Prediction(s)
Null	Uniform landscape	Isolation by Distance	No effect of landscape on gene flow
Land cover	Forest = low	Isolation by Resistance	Gaps in woodland (e.g., grassland/ treeless canyons) restrict movement and gene flow.
	Non-forest = high		
Elevation	< 1500 m = low	Isolation by Resistance	High elevations are a barrier to gene flow
	> 1500 m = high		
Land-elevation	Combined land cover and elevation resistance maps	Isolation by Resistance	1. Variation in elevation in combination with changes in forest composition (i.e. deciduous to coniferous) restricts gene flow
			2. Denser forests at high elevation facilitate gene flow
Hybrid	Pure zone = low	Isolation by Resistance	Poplar hybrid zones attract chickadees and thereby inhibit further gene flow
	Hybrid zone = high		

### Influence of landscape resistance on genetic distance

To test for an effect of pairwise landscape resistance distances on gene flow, each resistance distance matrix was compared with linearized pairwise genetic distance matrices (*F*_ST_ and *D*_EST_) using simple and partial Mantel tests in IBDWS v3.2.3 [64]. Statistical significance was determined by 10,000 permutations. Mantel tests were performed on all 15 populations for the four variables or distance matrices (null, land cover, elevation and land-elevation), and for 12 populations after incorporating the variable ‘hybrid’. Since sample sizes were not consistent across populations, we removed any sample size related bias by testing for the effect of landscape resistances on pairwise mean individual genotypic distances (GD; calculated in GenAlEx v6.5).

As Mantel tests do not account for non-independence of each pairwise observation within the distance matrix (i.e., each pairwise distance is associated with two different populations), they are assumed to have relatively low statistical power [77–79]. To overcome this drawback, we implemented a linear mixed-effect modelling approach using the ‘lmer’ function in the package ‘lmer4’ v 1.1.8 [80] in R [72] and the same pairwise landscape and genetic distances used in the Mantel analyses. This method is based on the maximum-likelihood population-effects (MLPE) model [81], developed to account for non-independence.

A strict model selection was conducted in two steps to test for the effects of different landscape (or explanatory) variables on pairwise genetic distances (i.e., *F*_ST_ and *D*_EST_). The same set of models were used as the Mantel tests to allow a direct comparison of model fit. First, models were tested for all 15 populations, followed by a separate model set after including the ‘hybrid’ parameter for 12 populations that were in or near hybrid zones. For each fitted MLPE model, the explanatory variables represented the fixed effect whereas the random effect, which remained constant, represented the dependency between pairwise distances (i.e., individual population effect). The ‘lmer’ function was modified so that the random factor would account for multiple memberships [82]. Prior to analyses explanatory variables were centred around their mean and models were checked for normality and multicollinearity. Parameter estimation was performed with the residual maximum-likelihood (REML) criterion [81] and 95% confidence intervals were calculated. Finally, we used the marginal *R* ^2^ statistic developed by [83] in the R package, MuMIn v 1.14.0 [84], to select the best fitting model.

## Results

### Genetic diversity and population structure

A total of 343 individuals from 28 locations were successfully genotyped for 12 variable microsatellite loci (Table 1). The number of alleles per locus ranged from 2–33 (S2 Table). Null alleles were detected in eight populations (with inconsistencies across populations) and the frequency was low with the exception of two loci: VeCr05 (0–70%) and Cuμ28 (0–73%). Large discrepancies between observed and expected heterozygosities were found for both loci (S3 Table), but these were not consistent across populations. We therefore carried out all additional analyses with and without those two loci for comparison, but as no considerable differences in results were observed, both VeCro05 and Cuμ28 were retained. Population LE contained the largest number of private alleles (*PA* = 10) followed by SB1 (*PA* = 5) (Table 1). Overall observed and expected heterozygosities ranged from 0.564 (RD1) to 0.714 (BUC), and 0.633 (BUC) to 0.708 (DR and CR; Table 1) respectively. Allelic richness ranged from 4.01 (SSK) to 4.79 (CR) and inbreeding coefficient from -0.122 (BUC) to 0.204 (RD1) (Table 1). After corrections for multiple tests, we found two deviations from HWE and three pairs of loci in disequilibrium. LE deviated from HWE at two loci; VeCr05 and Pij02 and significant LD was found between loci Titgata39 and CTC101 (*P* ≤ 0.001) within RD2 and between loci PAT MP 2–14 and Titgata39 (*P* ≤ 0.001) within populations SSK and LE (*P* ≤ 0.001).

Pairwise *F*_ST_ and *D*_EST_ values showed low to moderate genetic differentiation among population comparisons ranging from 0.007–0.049 (*F*_ST_) and 0.000–0.089 (*D*_EST_). Population wide *F’*_ST_ was 0.060. After corrections for multiple tests, 50 (*D*_EST_) and 52 (*F*_ST_) of the 105 tests were significant (Table 3). For *F*_ST_, three populations (LE, DR and SSK) were significantly differentiated from all other populations; two of which (LE and DR) are situated within a poplar hybrid zone. In addition, BO was significantly differentiated from all populations south of the Bow River. Significant pairwise *D*_EST_ values confirm these patterns. A standard AMOVA with no hierarchy generated an *F*_ST_ of 0.020 and 2.04% of the variance was among populations and 97.96% within populations (*P* ≤ 0.001). Using a hierarchical AMOVA, a number of groups were tested to identify the grouping that explains the largest among group variance (S4 Table). The highest among group variance (1.80%) was explained using two groups (SSK and all remaining populations), followed by two different groups (DR and all remaining populations; 1.11%). Interestingly, among group variance was also high when grouping was based on riparian poplar species (1.08%), but not when grouping by river system (0.08%). Finally, we found no significant effect of Euclidean distance on either *F*_ST_ (*R* ^2^ = 0.01, *P* = 0.106) or *D*_EST_ (*R* ^2^ = 0.02; *P* = 0.128).

**Table 3:** *F*_ST_ and *D*_EST_ estimates of population genetic differentiation.

	BUC	NSK	IN	RD1	RD2	DR^1^	BO^1^	SB2	DY	SB1	CR	OM^2^	FO^2^	LE^3^	SSK
BUC		**0.036**	0.008	0.001	0.006	0.038	**0.036**	0.004	0.007	0.000	0.007	**0.039**	0.005	**0.044**	**0.037**
NSK	0.031		0.015	0.012	0.026	**0.051**	0.012	0.012	0.016	**0.031**	**0.044**	**0.036**	0.020	**0.039**	**0.062**
IN	0.052	0.029		0.000	0.006	0.026	**0.050**	0.013	0.023	0.005	0.013	0.012	0.007	**0.062**	**0.021**
RD1	0.029	0.015	0.027		0.000	0.019	0.012	0.003	0.000	0.001	0.006	0.015	0.000	**0.026**	**0.027**
RD2	0.028	0.022	0.029	0.017		0.007	**0.047**	0.007	0.000	0.005	0.000	0.022	0.000	**0.023**	**0.043**
**DR** ^**1**^	**0.042**	**0.024**	**0.032**	**0.023**	**0.024**		**0.057**	**0.019**	**0.022**	**0.039**	**0.014**	0.059	0.012	**0.089**	**0.080**
**BO** ^**1**^	**0.035**	0.012	0.033	0.014	0.023	**0.025**		**0.013**	**0.028**	**0.029**	**0.041**	**0.087**	**0.032**	**0.044**	**0.092**
SB2	0.026	0.012	0.031	0.011	0.016	**0.025**	0.013		0.000	0.000	0.005	**0.042**	0.000	**0.036**	**0.047**
DY	0.027	0.017	0.034	0.015	0.016	**0.026**	**0.021**	0.010		0.003	0.001	0.028	0.001	**0.019**	**0.044**
SB1	0.026	**0.017**	0.030	0.010	**0.016**	**0.027**	**0.017**	0.007	0.014		**0.013**	**0.040**	0.006	**0.025**	**0.027**
CR	0.028	0.020	0.028	0.017	0.010	**0.022**	**0.021**	0.014	0.014	**0.017**		0.023	0.002	**0.047**	**0.046**
**OM** ^**2**^	0.039	**0.036**	0.049	0.033	0.026	**0.041**	**0.043**	**0.035**	0.030	**0.036**	0.028		0.008	**0.042**	**0.036**
**FO** ^**2**^	**0.035**	0.023	0.046	0.020	0.024	**0.042**	**0.028**	0.012	0.019	0.018	0.025	0.036		**0.015**	**0.052**
**LE** ^**3**^	**0.035**	**0.017**	**0.040**	**0.015**	**0.023**	**0.032**	**0.018**	**0.015**	**0.021**	**0.014**	**0.024**	**0.034**	**0.021**		**0.073**
SSK	**0.039**	**0.030**	**0.039**	**0.027**	**0.022**	**0.037**	**0.034**	**0.024**	**0.025**	**0.026**	**0.023**	**0.035**	**0.036**	**0.032**	

Pairwise *F*_ST_ values (below diagonal) and harmonic mean estimates of *D*_EST_ (above diagonal) for 15 black-capped chickadee populations based on 12 microsatellite loci. Bold values indicate statistical significance after FDR correction.

^1^Populations associated with *P*. *balsamifera* x *P*. *deltoides* poplar hybrid zone

^2^Populations associated with *P*. *balsamifera* x *P*. *angustifolia* poplar hybrid zone

^3^Populations associated with *P*. *balsamifera* x *P*. *angustifolia* x *P*. *deltoides* poplar hybrid zones (see Fig 1).

### Genetic clustering results

**Fig 2:**
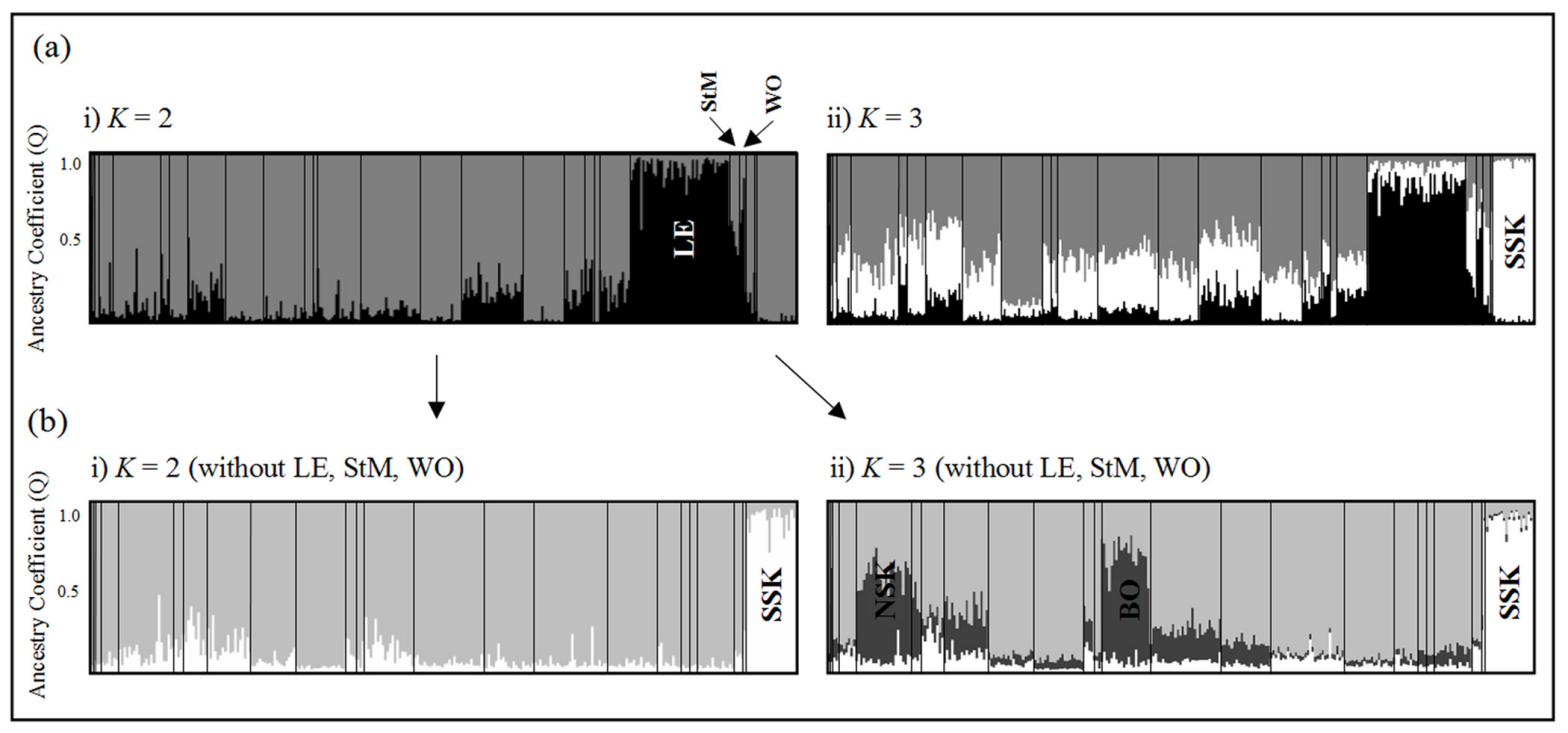
Individual assignment to *K* clusters based on the Bayesian clustering program, STRUCTURE. Inferred population structure of the black-capped chickadee (*Poecile atricapillus*) from 12 microsatellite loci. Runs were conducted for two values of *K*, but the optimal number of clusters to describe the data was unclear. The initial run (a) for all individuals from 28 localities resulted in contrasting values of true *K*: (i) *K* = 2 (Δ*K*) and ii) *K* = 3 (LnPr (X|K)). After choosing *K* = 2 as the true value, we removed the genetic cluster containing LE, StM and WO and did a second run (b) which produced contrasting results: (i) *K* = 2 (Δ*K*) and ii) *K* = 3 (LnPr (X|K)). Due to mixed assignment of NSK and BO at *K* = 3, we chose *K* = 2 as the true value. No additional structure was identified after removing population SSK. Overall, STRUCTURE identified three genetic clusters (cluster 1: LE, StM and WO; cluster 2: SSK and cluster 3: all remaining populations).

**Fig 3:**
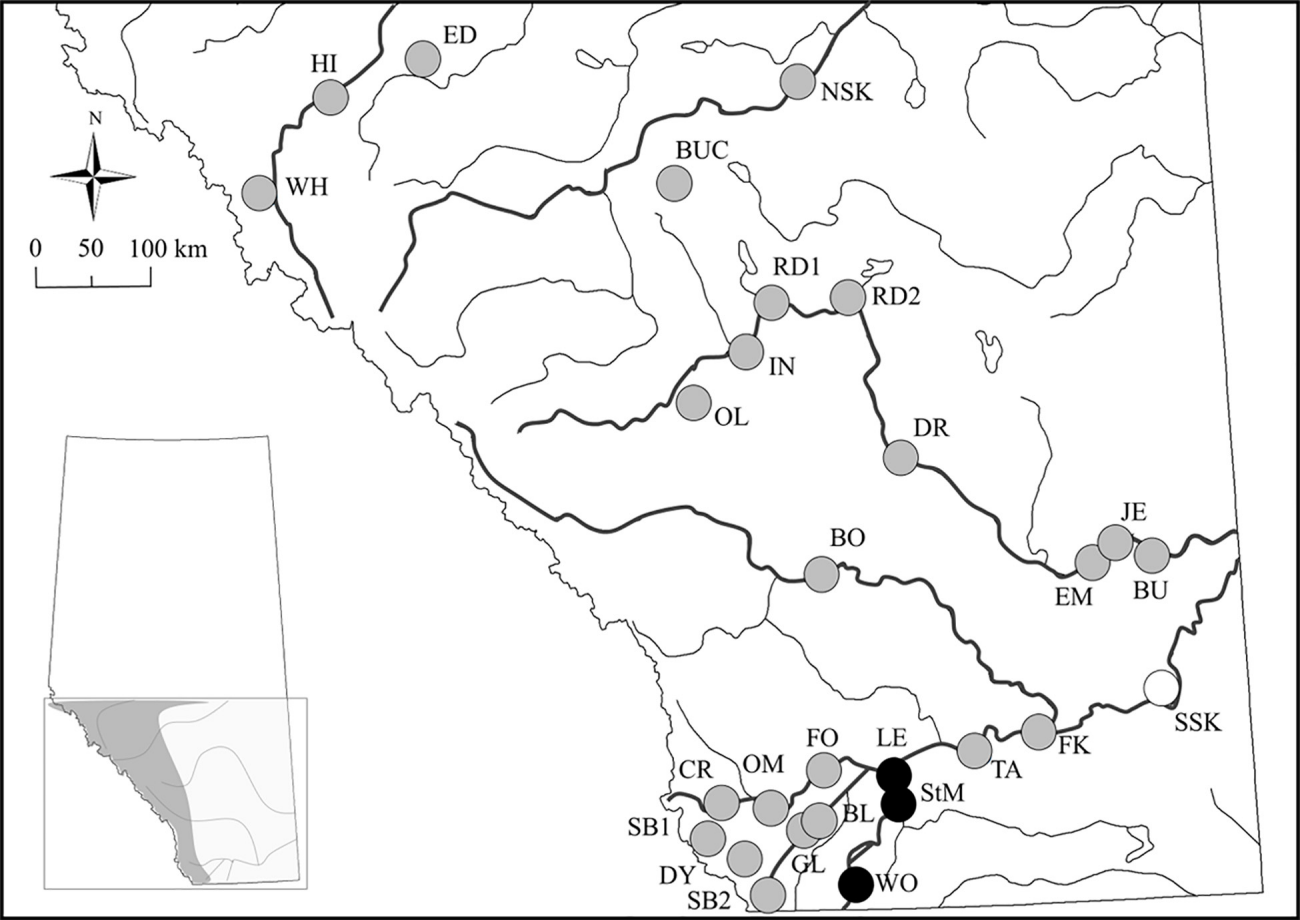
Inferred genetic clusters from STRUCTURE. Sampling locations (See Table 1 for abbreviations and associated river systems) with inferred clusters from STRUCTURE (coloured circles; *K* = 3; see Fig 2). Inset illustrates forest cover in the area (dark grey = forest; light grey = grassland).

All individuals were included in the structure run as clustering results should not be affected by small sample sizes. One exception may be when a small number of individuals from a genetically distinct populations are included, however, that does not appear to be the case in this study. Delta *K* (Δ*K*) and mean log likelihood (LnPr(X|R)) for the initial STRUCTURE runs involving all 343 individuals showed two (*K* = 2) and three (*K* = 3) groups respectively (Fig 2A; S1 Fig). Assignments for *K* = 3 had individuals with Q values suggesting mixed ancestry (Fig 2A (ii)) which implies oversplitting of populations, therefore, we chose *K* = 2 (Fig 2A (i)) as our true initial *K*. We then ran admixed individuals from StM and WO (Fig 2A (i)) with one pure population from each of the two clusters and confirmed that StM and WO individuals clustered with LE individuals. Using a hierarchical approach and removing the LE, StM and WO cluster, SSK formed a distinct cluster. Again, there was disagreement between Δ*K* (*K* = 2) and mean log likelihood (*K* = 3) over the true *K* (Fig 2B). For *K* = 3, clustering of populations BO and NSK is evident (Fig 2B (ii)), however, when these populations were run together with RD1 (to represent the large genetic cluster), STRUCTURE identified only one genetic group (*K* = 1) suggesting that splitting of BO and NSK was an overestimation and so we took *K* = 2 as the true value (Fig 2B (i)). Overall, STRUCTURE identified three genetic clusters (cluster 1: LE, StM and WO; cluster 2: SSK and cluster 3: all remaining populations; Fig 3).

**Fig 4:**
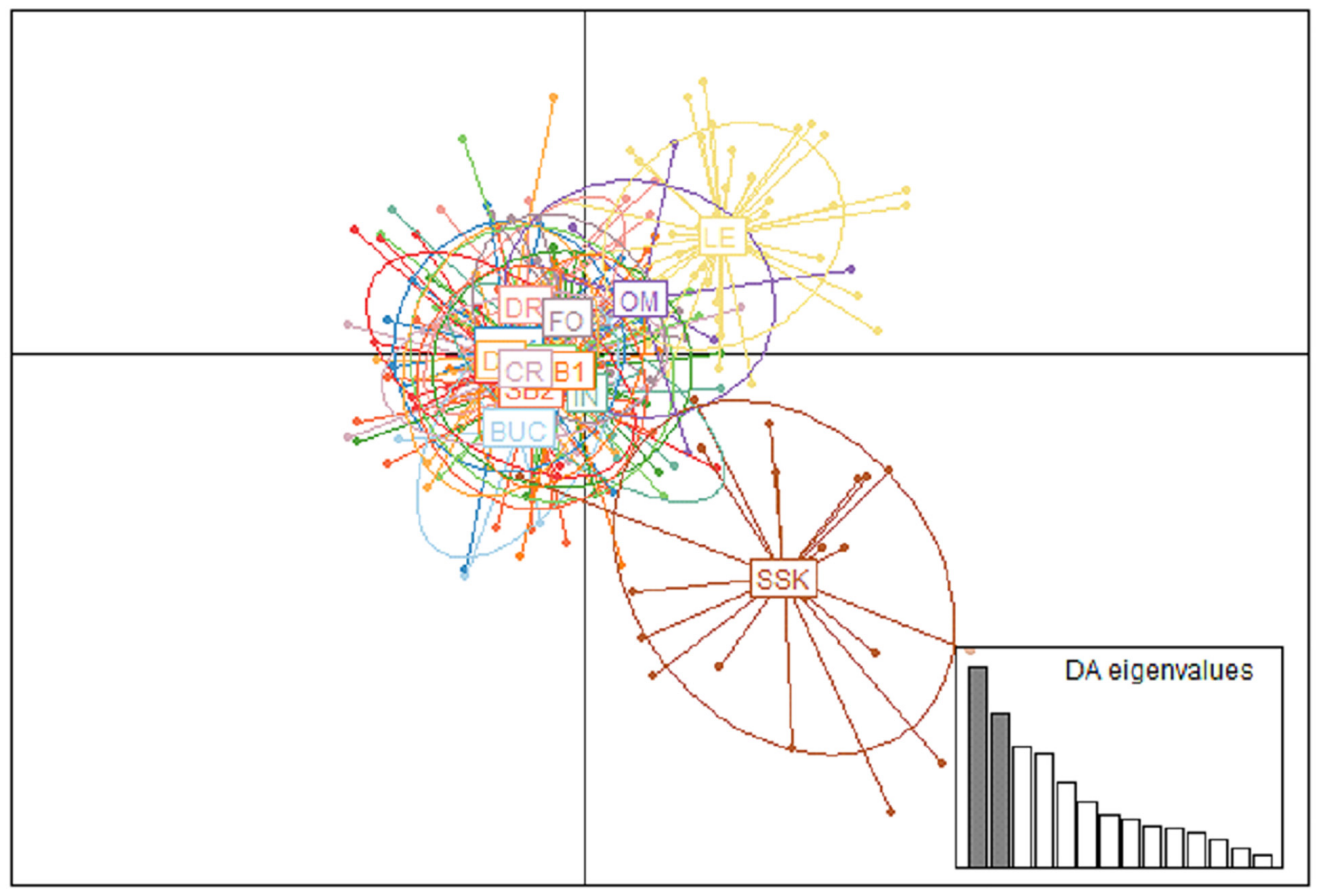
Genetic similarity inferred by discriminant analysis of principal components (DAPC). A representation of genetic relatedness between geographical clusters of black-capped chickadee populations (*N* = 15) obtained by DAPC. The graphs represent individuals as dots and the populations as inertia ellipses (population abbreviations can be found in Table 1) and scatterplots are based on the first two principal components. Populations with *N* ≤ 5 were excluded.

STRUCTURE analyses also showed that populations separated by natural gaps in riparian woodland were genetically differentiated from each other whereas those separated by artificial barriers were not (S5 Table). While these results are concordant with pairwise *F*_ST_ and *D*_EST_ analyses, populations separated by artificial barriers contained ≤ 10 individuals and additional sampling may yield different results. Similar to previous runs, LE and SSK are two genetically distinct populations (*K* = 2). In addition, STRUCTURE identified BO as genetically distinct from all southern populations (*K* = 2; for both LnPr (X|R) and Δ*K*) confirming significant *F*_ST_ values. DAPC analysis confirms population genetic differentiation in the black-capped chickadee. We see separation of SSK and LE on the x axis with some overlap (Fig 4); comparable with genetic structuring identified in STRUCTURE. DAPC failed to cluster StM and WO with LE and all other populations form one clearly defined cluster.

### Influence of the landscape resistance on genetic distance

All statistically significant Mantel correlations were positive (Table 4) and included the variables land cover (*r* = 0.59, *R* ^2^ = 0.35. *P* = 0.004) and land-elevation (*r* = 0.57, *R* ^2^ = 0.33, *P* = 0.006) for *F*_ST_, but only land cover (*r* = 0.38, *R* ^2^ = 0.15, *P* = 0.042) for *D*_EST_. There was no significant effect of isolation by distance, elevation or hybrid variables on either estimate of genetic distance. However, controlling for isolation by distance slightly increased the association between all five variables and *F*_ST_ in the partial Mantel tests, and between elevation and *D*_EST_. Controlling for land cover or elevation produced similar results.

**Table 4:** Summary of Mantel and partial Mantel tests.

Variable (controlled variable)	*F*_ST_		*D*_EST_	
	*r*	*R* ^2^	*r*	*R* ^2^
Null	-0.05	0.01	-0.01	0
Elevation	-0.35	0.12	-0.28	0.08
Elevation (null)	-0.37		-0.32	
Elevation (land cover)	-0.14		-0.15	
Land cover	0.59**	0.35	0.38**	0.15
Land cover (null)	0.60**		0.38**	
Land cover (elevation)	0.52**		0.29	
Land-elevation	0.57**	0.33	0.35	0.12
Land-elevation (null)	0.59**		0.35	
Hybrid	0.18	0.03	0.02	0
Hybrid (null)	0.35		-0.04	

Mantel results compare the effect of different resistance distances (variables) on genetic distance (*F*_ST_ and *D*_EST_) for all populations (*N* = 15; above dashed line) and populations located within hybrid poplar zones (*N* = 12; below dashed line). Controlled variable for partial Mantel tests is stated in brackets (e.g., (null) = controlled for isolation by distance). Results include *r* = partial coefficient, *R* ^2^ = coefficient of determination and ** indicates significant correlations (*P* ≤ 0.05).

We found no significant effect of landscape resistance distances on pairwise mean individual GD (results not shown) nor when populations with ≤ 20 individuals were removed. As GD are more sensitive to picking up the effects of more recent fragmentation in comparison to *F*_ST_ and *D*_EST_ [85,86], and given that populations on either side of recent artificial barriers were not structured, this result was unsurprising.

Our MLPE model results suggest that land cover has a strongest influence on patterns of population genetic differentiation in the black-capped chickadee, which is concordant with the Mantel test results. For models based on all 15 populations, the best fitting model included the explanatory variable land cover for both *F*_ST_ (*R* ^2^ (mar) = 0.456) and *D*_EST_ (*R* ^2^ (mar) = 0.130). In both cases, land cover had a positive effect on genetic distance (*F*_ST_ = 0.40 ± 0.04; *D*_EST_ = 0.04 ± 0.03) (Table 5). Land-elevation also showed a significant, positive effect and was ranked as the second best fitting model. There was no effect of either elevation or isolation by distance on genetic distance estimates. Of the MLPE models tested for 12 populations located in or close to the hybrid poplar zones, the best fitting model included land cover for both *F*_ST_ (*R* ^2^ (mar) = 0.451) and *D*_EST_ (*R* ^2^ (mar) = 0.247) (Table 5) where a positive effect was found (*F*_ST_ = 0.04 ± 0.01; *D*_EST_ = 0.05 ± 0.05). The variable land-elevation ranked second and, with one exception, there was no effect of the remaining variables. The hybrid variable was ranked third for both genetic distances and showed a positive effect for *F*_ST_ (Table 5).

**Table 5:** MLPE fitted model results on the effects of five landscape variables on genetic differentiation.

Response variable	Model	Null	Elevation	Land cover	Land-elevation	Hybrid	Marginal *R* ^2^
*15 populations*							
*F*_ST_	3	—	—	**0.40 ± 0.04**	—	n/a	0.456
	4	—	—	—	**0.03 ± 0.01**	n/a	0.365
	2	—	-2.74 ± 1.04	—	—	n/a	0.258
	1	0.00 ± 0.01	—	—	—	n/a	0.001
*D*_EST_	3	—	—	**0.04 ± 0.03**	—	n/a	0.130
	4	—	—	—	**0.04 ± 0.03**	n/a	0.089
	2	—	-1.26 ± 2.58	—	—	n/a	0.013
	1	0.00 ± 0.01	—	—	—	n/a	0.001
*12 populations*							
*F*_ST_	3	—	—	**0.04 ± 0.01**	—	—	0.451
	4	—	—	—	**0.03 ± 0.01**	—	0.372
	5	—	—	—	—	**0.00 ± 0.00**	0.031
	1	0.01 ± 0.01	—	—	—	—	0.006
	2	—	0.27 ± 1.68	—	—	—	0.003
*D*_EST_	3	—	—	**0.05 ± 0.05**	—	—	0.247
	4	—	—	—	**0.05 ± 0.03**	—	0.190
	5	—	—	—	—	0.00 ± 0.00	0.012
	1	0.01 ± 0.02	—	—	—	—	0.010
	2	—	0.21 ± 3.97	—	—	—	0.000

Four explanatory variables were fitted for MLPE models for 15 populations (above mid-rule), and five variables for 12 populations (below mid-rule) to test for the effects of landscape variables on two measures of genetic distance (*F*_ST_ = above dashed line and *D*_EST_ = below dashed line). Models were ranked based on marginal *R* ^2^. Explanatory variables that were not included in the fitted model are indicated by ‘—’ and variables that were not tested are indicated by ‘n/a’. Values are presented as regression slope estimates ± 95% confidence interval and have been converted to x10^-4^. Bold values indicate significance where 95% confidence intervals which do not overlap zero.

Overall, the MLPE models found a positive influence of land cover (for both model sets) and hybrid zones (for the 12 population model set) on population genetic differentiation, but no effect of isolation by distance or elevation. Models were ranked in the same order for both model sets (Table 5). The effect of land cover on genetic distance was consistent across models and produced relatively high *R* ^2^ values. Although the effect of land-elevation was positive when included in the model and Mantel tests, the lack of effect of elevation as a single parameter suggests that this positive effect stems from the influence of land cover alone. While no correlation between hybrid zones and genetic distance was found in the Mantel tests, there appears to some effect of hybrid poplar zones on *F*_ST_.

## Discussion

In this study, we examined how riparian woodlands influence patterns of population structure and genetic differentiation in black-capped chickadees. These forested woodlands are expected to act as corridors to allow organisms to disperse across large areas of unsuitable prairie grassland habitat and to maintain population and genetic connectivity in heterogeneous landscapes. Despite the small spatial scale of the study area, as well as the dispersal potential of this generalist species, we found that black-capped chickadee populations were genetically differentiated. The most concordant groups are SSK; LE, StM and WO; and all remaining populations. Additional analyses also identified DR and BO to be significantly differentiated from other populations (Table 3). These results suggest other factors are influencing movement along these linear features.

A significant effect of landscape resistance distances on genetic distance revealed that variation in landscape features influences population differentiation in chickadees. Both Mantel correlations and MLPE model selection indicated a significant association between land cover and population genetic differentiation, with little or no support for the effect of geographical distance or elevation. Given the fragmented nature of the study area (i.e. woodlands are patchily distributed along river systems) and the dependence of birds on riparian woodland for movement, this result supported our predictions. Considering all possible landscape factors influencing dispersal is essential in studies such as this as even small gaps (e.g., approximately 45 km between LE and FO) in continuous habitat have a significant impact on population differentiation in a generalist and widespread species.

### Dispersal within river systems

Patterns of genetic differentiation within river systems, particularly in the east (e.g., SSK), suggest that gaps in riparian corridors restrict dispersal and gene flow, and the positive effects of land cover on *F*_ST_ and *D*_EST_ supports this observation. The distribution of riparian woodland is influenced by survival, establishment and regeneration of riparian poplars and natural disturbance regimes (e.g., adequate river flows, flooding, channel shifting and climate) [87] which can result in large breaks in riparian woodland. SSK is an isolated island within the South Saskatchewan River as it contains large stretches of unforested river valleys upstream and downstream of the SSK site. Chickadees at the SSK site are genetically distinct from all other populations, supporting our prediction that large gaps in woodland can isolate populations and lead to differentiation. Similarly BO is isolated from other southern populations (Table 3; S5 Table) and no riparian woodland is present downstream for approximately 150 km. The size of gaps seems to play a role in dispersal, with gaps ≥ 100 km impeding gene flow. Large gaps in tree cover along rivers showed similar effects for a declining riparian specialist, the purple-crowned fairy-wren (*Malurus coronatus*), where functional isolation of populations from natural stretches of treeless river (~ 140 km) contributed to patterns of genetic differentiation [88]. Therefore, it would be interesting to see further studies test this using a controlled methodological approach in other areas.

Human-mediated disturbances have had a huge impact on the health and survival of riparian ecosystems, and consequently, declines in riparian woodland [12] and disruptions to riverine communities [34,89] have been observed. A number of gap crossing studies of forest-dependent birds show evidence of reduced movement by gaps ≤ 100 m in forest cover [90–93]; however, in our study, populations on either side of artificial barriers within a river system do not appear to be significantly different (S5 Table). A temporal lag may explain why we did not observe genetic differentiation between populations on either side of artificial barriers, as the introduction of some barriers may be too recent to impact spatial genetic structure. Landguth *et al*. [94] found that the time to detect a genetic signal after the establishment of a barrier was approximately 15 generations for Mantel’s *r* whereas for *F*_ST_, it was ten times longer. With the oldest reservoir built in 1951 (St. Mary River), and the average lifespan of chickadees being 1.5–3 years (although some individuals can live up to 12 years), it is possible that genetic differentiation is not yet detectable using *F*_ST_. Alternatively, the smaller samples sizes from these populations may have impeded the resolution [86]. While there was no significant effect of landscape features on pairwise mean individual GD, additional samples over a larger number of artificial barriers and a larger area would be able to determine if sample size is the issue or if insufficient time has lapsed. Genetic differentiation was more pronounced in the east in comparison to the west. This pattern coincides with a gradual elevational gradient sloping from 1200 m in the west to 600 m in the east [11]. Despite finding no effect of elevation on genetic differentiation, this gradient combined with differences in riparian environments (Rocky Mountains to foothills to semi-arid prairies), substrate type (coarse vs. fine) and climatic variability (precipitation and temperature) contributes to variation in ecoclimatic zones which in turn affects poplar spp. distributions. For example, the densely populated *P*. *balsamifera* and *P*. *angustifolia* are found in the Rocky Mountains and foothills in the west, whereas the sparsely distributed *P*. *deltoides* is found in semi-arid grasslands of the east [11]. This corresponds to differentiation of DR and SSK which are found in *P*. *deltoides* sections of the river, whereas the genetic cluster containing LE, StM and WO coincides with the distribution of *P*. *angustifolia* (Fig 3). Clinal variation in landscape, climate and vegetation may explain these genetic patterns with chickadees favouring denser poplar stands [87]. However, this will require more systematic sampling to confirm that poplar tree species correspond to patterns of genetic differentiation. Finally, artificial plantations of poplars are common in Southern Alberta to promote woodland replenishment, and one example of this occurs in Taber (population TA). This may explain the anomaly in our clustering analyses with individuals in TA (as well as one individual in FK) clustering with the large genetic group in STRUCTURE (grey cluster; Fig 1) instead of neighbouring genetic groups (i.e., LE, StM and WO, and SSK).

### Dispersal between river systems

Rivers that cross the plains are confined to coulees (or valleys) of varying depth, but coulees themselves are separated by large expanses of grassland and low shrubby vegetation with scattered depressions (i.e., ponds, marshes or lakes) where patches of forest sometimes exist. In this study area, black-capped chickadees would therefore need to disperse approximately 100 km across unsuitable habitat between river systems which, given their low dispersal potential, is highly unlikely. While some populations on different rivers systems showed signs of differentiation in the east (e.g., LE and DR; Table 3), populations in the west did not. This suggests that populations are connected upstream by the abundance of treed areas in the parkland and foothill regions. Similar patterns of landscape connectivity between river systems, but in a topographically complex landscape were found in the Pacific jumping mouse (*Zapus trinotatus*) [95]. The Rocky Mountains do not appear to impede dispersal between river systems. In fact, we found no effect of elevation on genetic differentiation in the Mantel tests or MLPE models, unless it was combined with land cover (land-elevation). It is unlikely that elevation contributed to the positive effects of the variable land-elevation on genetic differentiation and therefore we have discounted this variable as a contributing factor. Given this information, we can also refute the hypothesis that unsuitable habitat (pure coniferous stands) combined with interspecific competition with increasing elevation reduces population connectivity between river systems in support of the alternative hypothesis.

Agricultural practices and long term and intensive grazing along river valleys are becoming a serious concern for the health of riparian woodlands, as well as abundance and diversity of riparian bird communities [31]. These processes may have contributed to patterns of genetic differentiation between nearby river systems separated by large areas of agricultural fields in the study area. For example, the St. Mary and Waterton River are separated by highly modified agricultural areas and chickadee populations in these areas are genetically distinct (Fig 3). Even highly vagile migratory species such as the American robin (*Turdus migratorius*), brown thrasher (*Toxostoma rufum*) and loggerhead shrike (*Lanius ludovicianus*) have been shown to preferentially cross agricultural landscapes through connecting woodland corridors [96], highlighting the importance of natural corridors for dispersal.

### Dispersal in hybrid poplar zones

It has been widely recognised that hybridisation is important for plant speciation [97], but there has been increasing evidence of the importance of hybrid poplar zones in influencing the abundance [98,99], preference [22,23], performance [27] and genetic diversity [25,100] of organisms associated with these tree species. Poplar hybrids often differ in tree architecture, phenology and chemical defences from their parental species and these characteristics have contributed to differences in arthropod distributions and can drive population genetic differentiation [25,100–102]. If they can influence the evolution of dependent arthropods, then they also have the potential to impact a wide range of taxa within the riparian community (e.g., microbes and vertebrates) and thus have important ecological and evolutionary roles for dependent organisms. Can they then drive genetic differentiation in chickadee populations? With the exception of SSK, almost all of the significant pairwise genetic distances (*F*_ST_ and *D*_EST_) are poplar hybrid zone-associated chickadee populations (e.g., DR and LE). Forty of the 52 significant pairwise *F*_ST,_ and 37 of the 50 significant pairwise *D*_EST_ comparisons, included populations associated with a di- or tri-specific poplar hybrid zone (Table 3). In addition, the MLPE models and AMOVA analyses also showed an effect of these hybrid zones on genetic distance. Even STRUCTURE identified a cluster within the trispecific hybrid zone. The combined genetic data suggest that, as predicted, hybrid poplar zones (particularly those containing the species *P*. *deltoides*) may be influencing population genetic differences in chickadees. It is possible their movement decisions are due to their ecologically rich and diverse community favourable for insectivorous, cavity nesting birds; however, this will require a more rigorous sampling design to test this hypothesis.

## Conclusions

This study demonstrated the importance of assessing patterns of population genetic differentiation at small spatial scales as additional substructure may go undetected. Determining the effects of landscape features on microevolutionary processes can provide additional insights into the way organisms interact with their environment. Here, we found significant genetic structuring of a common, resident riparian species which was not observed at the rangewide scale, as well as a significant effect of landscape variables on patterns of genetic differentiation.

Overall, large expanses of prairie grassland and breaks within the riparian corridor are important factors influencing population genetic differentiation. Despite the assumption that forested corridors can facilitate dispersal among river systems in highly fragmented landscapes, spatial heterogeneity within these corridors can lead to genetic isolation. Other genetic differences that could not be explained by gaps in woodland, coincide with poplar species distributions or hybrid zones. As such, these hybrid zones may have important conservation implications by promoting population divergence in poplar-dependent organisms and therefore requires further investigation. This study has demonstrated the need to explore genetic and environmental relationships at small geographical scales as understanding the role of landscape features on the genetic diversity of populations is crucial in helping to maintain genetic mixing and biodiversity.

## Supporting Information

S1 Fig Determining the correct number of genetic clusters in STRUCTURE. (a) Log likelihood plots (LnPr (X|K)) and (b) Δ*K* over *K* for STRUCTURE runs as shown in Fig 2. The most likely number of populations *K* is determined by the highest estimated log probability of the data and delta *K* infers the correct number of clusters from the difference of LnPr (X|K).
(DOC)

S1 Table Details of black-capped chickadee samples used in analyses. Sample ID’s in grey were removed from analyses.
(XLS)

S2 Table Microsatellite loci information. Repeat type (if known), primer sequence, allele size range (bp), number of alleles (***Na***) and MgCl2 concentration for each microsatellite locus used to genotype black-capped chickadee individuals.
(XLS)

S3 Table Microsatellite diversity measures. Table includes expected (*He*) and observed (*Ho*) heterozygosities and the total number of alleles (*Na*) for 15 populations of black-capped chickadees at 12 microsatellite loci. Overall population and loci means are also provided. See Table 1 for sampling site abbreviations.
(XLS)

S4 Table Hierarchical analysis of molecular variance. Table shows the percentage of variation for each of the three levels (among groups, among populations within groups and within populations) and across different group combinations. (ns = not significant; * = *P* ≤ 0.001)
(XLS)

S5 Table Structure barrier test. Results from STRUCTURE analysis of individuals from populations separated by artificial and/ or natural barriers to gene flow within river systems.
(XLS)
