# Supplementary material for: Gene Flow of a Forest-Dependent Bird across a Fragmented Landscape
Source: PLoS One. 2015 Nov 18;10(11):e0140938. doi: 10.1371/journal.pone.0140938 (PMC4651334; doi:10.1371/journal.pone.0140938)
Supplement: S1 Fig — (a) Log likelihood plots (LnPr (X|K)) and (b) ΔK over K for STRUCTURE runs as shown in Fig 2. The most likely number of populations K is determined by the highest estimated log probability of the data and delta K infers the correct number of clusters from the difference of LnPr (X|K). (DOC) [file pone.0140938.s001.doc]

1. i)

1. i)

ii)

ii)
